# Neuroinflammation and structural injury of the fetal ovine brain following intra-amniotic *Candida albicans* exposure

**DOI:** 10.1186/s12974-016-0492-z

**Published:** 2016-02-02

**Authors:** Daan R. M. G. Ophelders, Ruth Gussenhoven, Martin Lammens, Benno Küsters, Matthew W. Kemp, John P. Newnham, Matthew S. Payne, Suhas G. Kallapur, Allan H. Jobe, Luc J. Zimmermann, Boris W. Kramer, Tim G. A. M. Wolfs

**Affiliations:** Department of Pediatrics, Maastricht University Medical Center, PO box 5800, Maastricht, 6202 AZ The Netherlands; School of Mental Health and Neuroscience, Maastricht University, Universiteitssingel 40, Maastricht, 6229 ER The Netherlands; Department of Pathology, Antwerp University Hospital, Wilrijkstraat 10, 2650 Edegem, Belgium; Department of Pathology, Maastricht University Medical Center, PO box 5800, Maastricht, 6202 AZ The Netherlands; School of Women’s and Infants’ Health, The University of Western Australia (M550), 35 Stirling Highway, Crawley, WA 6009 Australia; Division of Neonatology/Pulmonary Biology, The Perinatal Institute, Cincinnati Children’s Hospital Medical Center, 3333 Burnet Ave., Cincinnati, OH 45208 USA; School of Oncology and Developmental Biology, Maastricht University, Universiteitssingel 50, Maastricht, 6229 ER The Netherlands

**Keywords:** Chorioamnionitis, *Candida albicans*, Fluconazole, Inflammation, White matter injury, Fetus, Preterm

## Abstract

**Background:**

Intra-amniotic *Candida albicans* (*C. Albicans*) infection is associated with preterm birth and high morbidity and mortality rates. Survivors are prone to adverse neurodevelopmental outcomes. The mechanisms leading to these adverse neonatal brain outcomes remain largely unknown. To better understand the mechanisms underlying *C. albicans*-induced fetal brain injury, we studied immunological responses and structural changes of the fetal brain in a well-established translational ovine model of intra-amniotic *C. albicans* infection. In addition, we tested whether these potential adverse outcomes of the fetal brain were improved in utero by antifungal treatment with fluconazole.

**Methods:**

Pregnant ewes received an intra-amniotic injection of 10^7^ colony-forming units *C. albicans* or saline (controls) at 3 or 5 days before preterm delivery at 0.8 of gestation (term ~ 150 days). Fetal intra-amniotic/intra-peritoneal injections of fluconazole or saline (controls) were administered 2 days after *C. albicans* exposure. Post mortem analyses for fungal burden, peripheral immune activation, neuroinflammation, and white matter/neuronal injury were performed to determine the effects of intra-amniotic *C. albicans* and fluconazole treatment.

**Results:**

Intra-amniotic exposure to *C. albicans* caused a severe systemic inflammatory response, illustrated by a robust increase of plasma interleukin-6 concentrations. Cerebrospinal fluid cultures were positive for *C. albicans* in the majority of the 3-day *C. albicans*-exposed animals whereas no positive cultures were present in the 5-day *C. albicans*-exposed and fluconazole-treated animals. Although *C. albicans* was not detected in the brain parenchyma, a neuroinflammatory response in the hippocampus and white matter was seen which was characterized by increased microglial and astrocyte activation. These neuroinflammatory changes were accompanied by structural white matter injury. Intra-amniotic fluconazole reduced fetal mortality but did not attenuate neuroinflammation and white matter injury.

**Conclusions:**

Intra-amniotic *C. albicans* exposure provoked acute systemic and neuroinflammatory responses with concomitant white matter injury. Fluconazole treatment prevented systemic inflammation without attenuating cerebral inflammation and injury.

**Electronic supplementary material:**

The online version of this article (doi:10.1186/s12974-016-0492-z) contains supplementary material, which is available to authorized users.

## Background

Preterm birth is associated with chorioamnionitis which is defined as inflammation of the fetal membranes and amniotic fluid caused by microbial invasion [1, 2]. The microorganisms most frequently associated with this condition include *Ureaplasma species*, *Mycoplasma hominis*, and *Gardnerella vaginalis*, all of which most commonly originate from the lower reproductive tract [3]. These microorganisms and/or inflammatory mediators in the amniotic cavity can cause a fetal inflammatory response syndrome (FIRS) [4–6]. Chorioamnionitis and subsequent FIRS are independent risk factors for adverse outcomes, including injury of the fetal brain [5, 7]. Adverse neurodevelopmental outcomes result from diffuse cerebral inflammation and white matter injury, periventricular leukomalacia, and intraventricular hemorrhage [5, 8, 9]. These conditions are associated with a high mortality rate, and survivors are predisposed to long-term morbidity including mental retardation, impaired learning, visual disorders, and in severe cases, cerebral palsy [7, 8, 10].

The pathophysiology of chorioamnionitis can also include viral and fungal species [4, 11, 12]. *Candida albicans* (*C. albicans*) is a commensal fungus of the gastro-intestinal tract which can be asymptomatic in the vaginal microbiota with increasing incidence during pregnancy [13, 14]. Intra-amniotic *C. albicans* infections are associated with high mortality rates and severely impaired neurodevelopmental outcomes [13–16] in which the mechanisms linking fetal exposure to neurological pathologies remain essentially unstudied. We hypothesized that antenatal exposure to *C. albicans* caused a neuroinflammatory response and subsequent white matter injury, which we tested by exposing fetal sheep to intra-amniotic *C. albicans* [16, 17].

In two clinical cases, intra-amniotic *C. albicans* infections resolved after oral and intra-amniotic fluconazole (F) [13]. Fluconazole is the most frequently used antifungal in *C. albicans* infections, and inhibits ergosterol synthesis, which is an essential component of fungal cell membranes [18]. We therefore further hypothesized that fetal intra-amniotic and intra-peritoneal administration of fluconazole would reduce the neuroinflammatory response and subsequent white matter injury to the fetal brain. Accordingly, systemic immune activation, neuroinflammation, and structural white matter injury were assessed in the fetal sheep exposed to intra-amniotic *C. albicans* and treated with fluconazole.

## Methods

### Experimental design

**Fig. 1:**
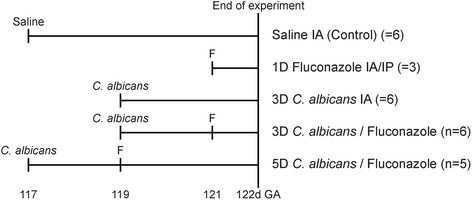
Experimental setup. Animals were exposed to *C. albicans* for 3 or 5 days in the absence or presence of intra-amniotic (IA)/intraperitoneal (IP) fluconazole (F). Control animals received intra-amniotic (IA) injection with saline. Fetuses were delivered at 0.8 of gestation

The study was approved by and performed according to the guidelines of the animal ethics committee of the University of Western Australia (Perth, Australia). Twenty-six date-mated Merino ewes with singleton pregnancies were randomly assigned to receive an intra-amniotic injection of saline (2 mL) as a control or *C. albicans* (10^7^ colony-forming units [CFU], Western Australian clinical isolate) [16]. After 2 days, an intra-amniotic/-peritoneal injection of fluconazole (30 mg per injection, Claris Life Sciences Limited, Chacharwadi-vasana, Ahmedabad-382 213, India) or saline (controls) was administered with delivery after 1 and 3 days (Fig. 1). Intra-amniotic injections were performed as previously described [17].

We did not include a 5-day *C. albicans-*only group since previous results with this model indicated that 5 days exposure to *C. albicans* alone was lethal [16, 17]. Given the survival of the 5-day *C. albican*s-/fluconazole-treated group, we have concluded that fluconazole in this model increases survival [16, 17].

### Tissue collection

Fetuses were delivered by Cesarean section at 0.8 of gestation (term ~150 days) [19], equivalent of 32–34 weeks human gestation [20], and euthanized with intravenous pentobarbitone (100 mg/kg). Amniotic fluid, blood, and cerebrospinal fluid (CSF) were collected at delivery, and cultures for *C. albicans* were performed [16]. Briefly, amniotic fluid (100 μL) was inoculated onto Difco Sabouraud Dextrose agar and incubated at 37 °C for 48 h. Blood (2 mL) and liquor (1 mL) were inoculated into BACTEC Peds Plus/F culture vials (Becton Dickinson, Franklin Lakes, NJ) and incubated aerobically at 37 °C for 72 h [16]. Brains were immersion-fixated in 4 % paraformaldehyde.

### Enzyme-linked immunosorbent assay interleukin-6

Plasma interleukin-6 (IL-6) concentrations were assessed as an indication for systemic inflammation using a sheep-specific sandwich enzyme-linked immunosorbent assay (ELISA). First, 96-well plates were pre-coated with 100 μL of mouse anti-ovine monoclonal antibody (MAB1004, Millipore, Darmstadt, Germany, working concentration 1:200) overnight at 4 °C. Plates were washed three times with 0.05 % Tween-20 in phosphate-buffered saline (PBST). Non-specific binding was blocked with a blocking buffer (1 % bovine serum albumin [BSA] in PBST) for 1 h followed by a washing step.

Protein standards were prepared by serial dilution of recombinant IL-6 (ImmunoChemistry Technologies, Bloomington, MN, USA). Plasma samples (100 μL) were loaded per well in duplicate and incubated for 2 h at room temperature. Unbound protein was omitted by washing with PBST, followed by incubation with detection antibody (rabbit anti-ovine IL-6, AB1839, Millipore, Darmstadt, Germany, working concentration 1:500) for 60 min and subsequent washing. Next, each well was incubated for 30 min with 100 μL of a goat anti-rabbit HRP (Jackson ImmunoResearch Laboratories Inc, West Grove, PA, USA, working concentration 1:500) followed by washing and incubation with 3,3′,5,5′-tetramethylbenzidine (TMB) substrate solution for 15 min at room temperature. The reaction was stopped by addition of 50 μL 2N sulfuric acid to each well. Plates were then read on an ELISA plate reader at 450 nm.

### Histology and immunohistochemistry

The right hemisphere was divided into four defined anatomical regions. Serial coronal sections of identical thickness (4 μm) of the region containing the posterior hippocampus and mid-thalamus were cut with a Leica RM2235 microtome. Within this region, we analyzed the hippocampus and the cerebral white matter since these regions are most affected following intra-uterine infection at this developmental stage [21]. All stainings were performed in series (every 15th section), and four sections per staining per animal were analyzed. Hematoxylin and eosin (H&E) and periodic acid-Schiff (PAS) stainings were performed for morphological and anatomical analysis and for identification of pseudohyphae of *C. albicans*, respectively. Adjacent sections were stained for ionized calcium-binding adaptor molecule 1 (IBA-1) (Wako Pure Chemical Industries, Osaka, Japan) for microglia, glial fibrillary acidic protein (GFAP) (DAKO Z0334) for astrocytes, myelin basic protein (MBP) (Merck Millipore, MAB386) for myelin sheaths, 2',3'-cyclic-nucleotide 3'-phosphodiesterase (CNPase) (Sigma, C5922) for mature myelin-producing oligodendrocytes, oligodendrocyte transcription factor 2 (Olig2) (Millipore, AB9610) for oligodendrocyte lineage cells, cluster of differentiation (CD) 68 (DAKO, M0718) for active microglia/phagocytizing macrophages, myeloperoxidase (MPO) (DAKO, A0398) for neutrophils, CD3 (DAKO A0452) for T-lymphocytes, Ki67 (DAKO, M7240) for cell proliferation, and cleaved caspase-3 (cell signaling, #9661) for apoptosis.

Sections were deparaffinized and rehydrated. Endogenous peroxidase activity was inactivated with 0.3 % H_2_O_2_ treatment. For every immunolabeled stain with the exception of GFAP and CD68, antigen retrieval was performed by microwave boiling of tissue sections in citrate buffer, pH 6.0. For CD68, antigen retrieval was performed with proteinase K (DAKO, S3004) treatment for 5 min at 37 °C. Nonspecific binding was blocked by incubation with 5 % BSA (MBP, Olig2, and CD3) or goat serum in phosphate-buffered saline (PBS) (IBA-1, GFAP, CNPase, CD68, MPO, and Ki67). Sections were incubated overnight at 4 °C in a closed humidity chamber with primary antibody (IBA-1, GFAP, MBP, CNPase, and caspase-3 1:1000; Olig2, MPO, and CD3 1:200; Ki67 1:100; CD68 1:50) and subsequently incubated with the specific secondary antibody. Immunostaining was enhanced with a Vectastain ABC peroxidase Elite kit (Vector Laboratories Inc, Burlingame, CA) followed by a (nickel) 3,3'-diaminobenzidine (DAB) staining and 0.1 % Nuclear Fast Red (Olig2, CD3, Ki67, and caspase-3) or Mayer’s hematoxylin (CD68 and MPO) for background staining. Sections were dehydrated and cover-slipped.

To identify the mature and total number of oligodendrocytes, an immunohistochemical procedure for sequential double staining of CNPase and Olig2 was applied. In this procedure, sections were treated as described above and incubated with the anti-CNPase antibody, followed by incubation with a biotinylated anti-mouse secondary antibody and visualization with 3,3'-diaminobenzidine (DAB; Dako). Before incubation with the Olig2 primary antibody, slides were blocked again with 5 % BSA for 60 min. Cells that stained positive for Olig2 were visualized by HistoGreen (Linaris, E109).

### Immunofluorescence

To identify *C. albicans* in the brain tissue, immunofluorescence staining was performed as described previously [17] using a rabbit anti-*C. albicans* antibody (Meridian Life Science, Memphis, TN, working concentration 1:50) and appropriate Alexa Fluor 594-labeled secondary antibody (working concentration 1:200). Briefly, sections were deparaffinized, rehydrated, and incubated in 0.25 % ammonia dissolved in 70 % ethanol for 1 h on a shaker. Antigen retrieval was performed using citrate buffer (pH 6.0) in a microwave oven. Nonspecific binding was blocked by incubation with 10 % goat serum for 1 h followed by overnight incubation with primary anti-*C.albicans* antibody dissolved in 0.1 % PBS with 0.2 % Tween-20. For fluorescence staining, Alexa Fluor-conjugated secondary antibody was used followed by incubation in 0.3 % Sudan Black Solution to reduce auto-fluorescence. Counterstaining was done with the fluorescent nuclear marker Hoechst.

To assess neuronal injury, a Fluoro-Jade C staining was performed, which is a specific marker for identification of degenerating neurons [22, 23]. In this procedure, sections were deparaffinized, rehydrated, and immersed in 0.06 % potassium permanganate with 0.01 % Hoechst for 10 min. Before immersing in the Fluoro-Jade C solution, sections were rinsed in running tap water for 1–2 min. Immersion in the Fluoro-Jade C solution (Millipore, AG325) (0.01 % stock solution in 0.1 % acetic acid) was performed in the dark for 10 min. Sections were washed with distilled water and subsequently air-dried at 50 °C. Finally, sections were cleared with xylene for 1 min before being cover-slipped using DPX.

### Qualitative analysis

H&E-stained sections were analyzed by three independent investigators and two neuropathologists, blinded to the experimental groups, to assess overall brain structure and inflammatory changes. The absence of tissue autolysis in our sections was confirmed by neuropathologists. The sections were examined for the presence of hemorrhages, gliosis, (cytotoxic) edema, abscess formation, and structural damage, including cyst formation. *C. albicans* fluorescent and PAS-stained sections were examined for the presence of *C. albicans*.

### Quantitative analysis

For the analysis of IBA-1, GFAP, MBP, CNPase, Olig2, and CD68 immunoreactivity, digital images of the hippocampus, white matter (WM), and periventricular white matter (PVWM) were acquired using an Olympus AX-70 microscope (Olympus, Tokyo, Japan) equipped with a black and white digital camera. From each section, one picture of the hippocampus was taken at a ×20 magnification, and images in the WM were taken at a ×100 magnification of the gliotic foci and 4–6 consecutive images of the PVWM. The area fraction of IBA-1, GFAP, and MBP immuno-reactivity was determined with a standard intensity threshold to determine positive staining using QWin Pro V 3.5.1 software (Leica, Rijswijk, The Netherlands). Blood vessels and artifacts were excluded from analysis. The CNPase- and Olig2-positive cells were counted using QWin software and expressed as cells/mm^2^. Ki67-, caspase-3-, CD3-, and MPO-positive cells were counted in all brain regions, focusing on the cerebral vasculature. The digital images were acquired and analyzed by an independent observer who was blinded to the experimental groups. Fluoro-Jade C/Hoechst stainings were examined using the Olympus AX-70 fluorescent microscope.

### Data analysis

Statistical analysis was performed with GraphPad Prism software (version v5.0; GraphPad Software Inc., La Jolla, CA, USA). Results were analyzed using the non-parametric Kruskal-Wallis test with Dunnett’s post-hoc testing to compare different groups. Results are given as mean and standard error of the mean (SEM) with significance at *p* < 0.05.

## Results

### *C. albicans* culture

*C. albicans* colonial morphology was confirmed by growth on Brilliance Candida Agar (Oxoid, Adelaide, Australia). Positive cerebrospinal fluid (CSF) cultures for *C. albicans* were detected in 67 % (4/6) of the 3-day *C. albicans*-exposed animals and in 50 % (3/6) of the 3-day *C. albicans-*/fluconazole-treated animals (Table 1). No positive cultures were present in the 5-day *C. albicans-*/fluconazole-treated group (0/5). Control animals had negative CSF cultures. Results of amniotic fluid and blood cultures were previously published by Maneenil and colleagues and are also summarized in the table [16].

**Table 1:** *Candida albicans* cultures

	Control	3-day *C. albicans*	3-day *C. albicans* and fluconazole	5-day *C. albicans* and fluconazole
Cerebrospinal fluid	0/6	4/6	3/6	0/5
Fetal blood^a^	0/6	4/6	5/6	3/5
Amniotic fluid^a^	0/6	6/6	6/6	5/5

The ratio of *C. albicans*-positive/total cultures are depicted

^a^Based on previously published data [16]

### No evidence of *C. albicans* invasion in the brain

Despite substantial numbers of animals with positive CSF cultures for *C. albicans*, PAS and immunofluorescent staining did not identify *C. albicans* in the parenchyma of the brains of any of the experimental groups (data not shown).

### Pro-inflammatory cytokine response

**Fig. 2:**
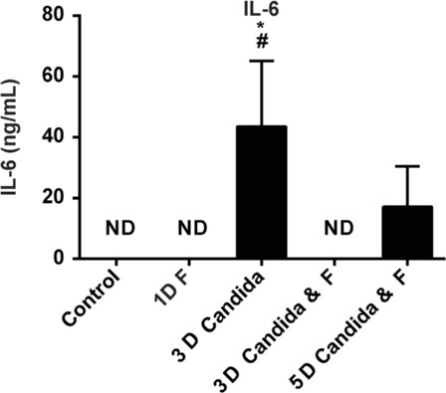
Plasma IL-6 concentrations following intra-amniotic *C. albicans* and/or fluconazole treatment. For statistical analysis, undetectable values were assigned an arbitrary value of 1 pg/mL. *p ≤ 0.05 vs. control; ^#^ *p* ≤ 0.05 vs. 3-day *C. albicans*/fluconazole

Significantly elevated plasma IL-6 concentrations were found in 50 % (3/6) of the 3-day *C. albicans*-exposed animals when compared to controls (Fig. 2). Forty percent (2/5) of the 5-day *C. albicans-*/fluconazole-treated animals demonstrated elevated plasma concentrations of IL-6; however, statistical significance was not reached. Plasma IL-6 concentrations in the controls, 1-day fluconazole- and 3-day *C. albicans-*/fluconazole-treated animals, were not detectable.

### Qualitative analysis of the brain

**Fig. 3:**
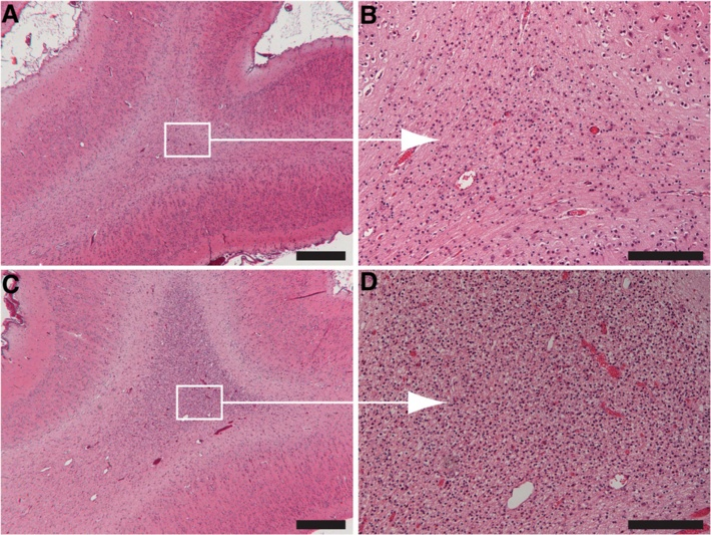
H&E-stained brain sections of control animals and 5-day *C. albicans-*/fluconazole-treated animals. Control animals (**a**, **b**) showed no to mild gliosis, and the 5-day *C. albicans-*/fluconazole-treated animals (**c**, **d**) showed severe gliotic foci in white matter of the gyral crest. **a** and **c** at ×25 magnification (scale bar = 500 μm) and **b** and **d** at ×200 magnification, *scale bar* = 100 μm

Qualitative analysis of H&E-stained sections revealed no evidence of structural changes including intraventricular hemorrhages and cystic lesions in any of the experimental groups. However, the brains of *C. albicans*-exposed animals had increased cell density in the white matter which primarily consisted of glial cells and blood-derived macrophages, that cannot be distinguished from microglia (Fig. 3). These gliotic lesions were primarily present in the gyral crests of the white matter and were more pronounced in 100 % (5/5) of animals of the 5-day *C. albicans-*/fluconazole-treated group (Fig. 3c, d) and in 67 % (8/12) of animals in the 3-day *C. albicans*-exposed groups. Control animals had mild to no gliotic lesions (Fig. 3a, b). Interestingly, the subcortical white matter was not affected (Fig. 3c).

### Antenatal exposure to *C. albicans* caused microglia/macrophage and astrocyte activation in the hippocampus and white matter

**Fig. 4:**
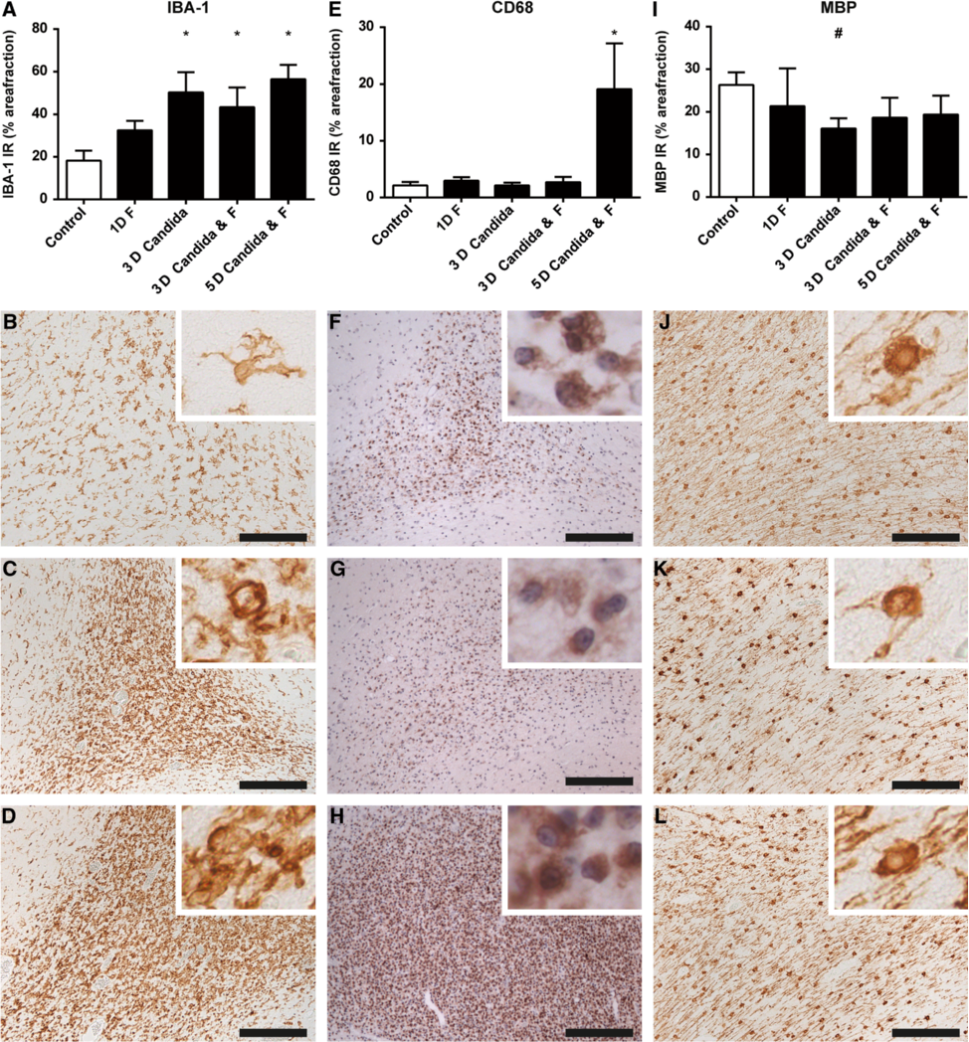
Antenatal exposure to *C. albicans* caused microglia activation and subsequent myelin disturbances in the white matter. Significant increase (*p < 0.05) of the area fraction of IBA-1 (**a**–**d**) immunoreactivity (IR) was observed in all *C. albicans*-exposed animals compared to controls irrespective of fluconazole (F) treatment. Significant increase (*p < 0.05) of the area fraction of CD68 IR (**e**–**h**) was observed in animals of the 5-day *C.* albicans/fluconazole group. A decrease of MBP IR (**i**–**l**) was observed which did not reach significance (^#^ *p* < 0.1) in the 3-day *C. albicans*-exposed group compared to the controls irrespective of fluconazole treatment. Representative figures show that the area fraction of the IBA-1 IR is higher in the 3-day *C. albicans* group (**c**) and 5-day *C. albicans-*/fluconazole-treated group (**d**) compared to the control group (**b**). Morphological analysis showed a higher density of amoeboid microglia present in the white matter after *C. albicans* exposure (*inserts*). In these regions with microglia activation, myelin disturbances, and loss of myelin fibers were found in the 3-day *C. albicans*-exposed (**g**) and the 5-day *C. albicans-*/fluconazole-treated (**h**) group. Figures of the 3-day *C. albicans-*/fluconazole-treated group are not depicted here. Images taken at **×**100 magnification (*insert* at **×**400 magnification), *scale bar* = 500 μm

**Fig. 5:**
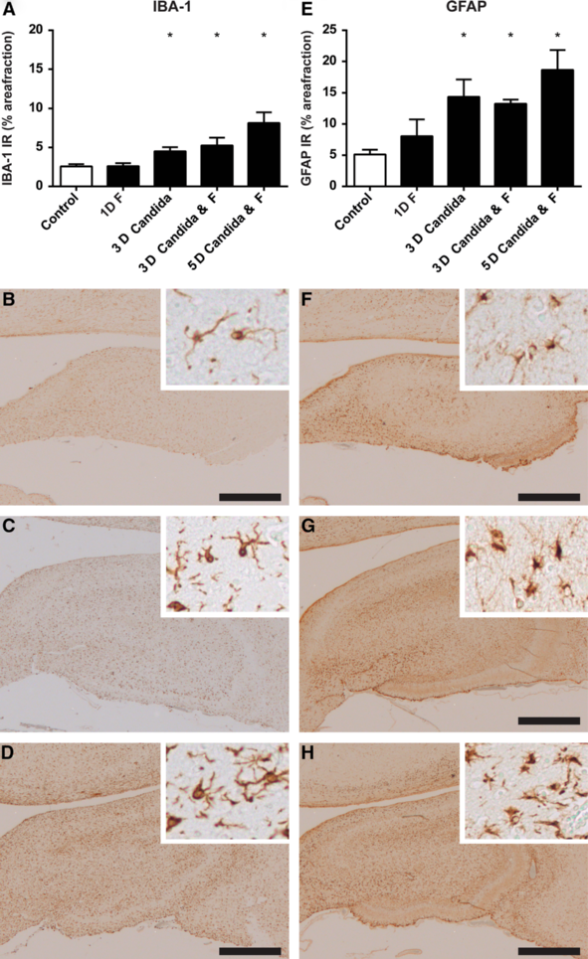
Antenatal exposure to *Candida albicans* caused microglial (IBA-1) and astrocyte (GFAP) activation in the hippocampus. A significant increase (*p < 0.05) of the area fraction of IBA-1 (**a**–**d**) and GFAP (**e**–**h**) immunoreactivity (IR) was observed in all *C. albicans*-exposed animals compared to controls irrespective of fluconazole (F) treatment (**a**, **e**). No significant changes were found in the fluconazole-treated animals compared to controls. Representative figures show that the area fraction of the IBA-1 IR and GFAP IR was higher in the 3-day *C. albicans* group (**c**, **g**) and 5-day *C. albicans-*/fluconazole-treated group (**d**, **h**) compared to the control group (**b**, **f**). Images at **×**20 magnification (*inserts* at **×**200 magnification), *scale bar* = 100 μm

The neuroinflammatory changes, as indicated by the more pronounced presence of gliotic foci, in *C. albicans*-exposed animals were further evaluated by immunohistochemical analysis of the microglial marker IBA-1. This staining demonstrated IBA-1 immunoreactivity (IR) distributed throughout the white matter in distinct foci in the *C. albicans-*exposed animals. These foci were primarily detected at the gyral crests of the white matter which corresponds with the increased cell density found in the H&E staining. This finding is substantiated by an increase of IBA-1 IR within these foci after intra-amniotic exposure to *C. albicans* irrespective of fluconazole treatment compared to the control group and/or 1-day fluconazole group (Fig. 4a–d). These foci comprised microglia of both ramified and amoeboid morphology. However, quantification of microglial activation based on microglial morphology was hampered by the high cell density within these foci. Immunoreactivity of CD68, representative for activated microglia/phagocytizing macrophages [24], was increased in the 5-day *C. albicans*/fluconazole group compared to controls (Fig. 4e–h). No significant changes in IBA-1 immunoreactivity in the periventricular white matter were observed. However, in 17 % (1/6) of the 3-day *C. albicans* animals and 60 % (3/5) of the 5-day *C. albicans*/fluconazole animals, foci containing CD68-positive cells were found in the periventricular white matter (data not shown). Besides an increase in IBA-1 immunoreactivity, an increase of the astrocyte marker glial fibrillary acidic protein (GFAP) was found in the hippocampus after intra-amniotic exposure to *C. albicans* compared to the control group and/or 1-day fluconazole group (Fig. 5). Administration of fluconazole irrespective of pre-exposure to *C. albicans* did not induce changes in IBA-1 or GFAP immunoreactivity.

### Antenatal exposure to *C. albicans* caused focal white matter disturbances with loss of mature oligodendrocytes

**Fig. 6:**
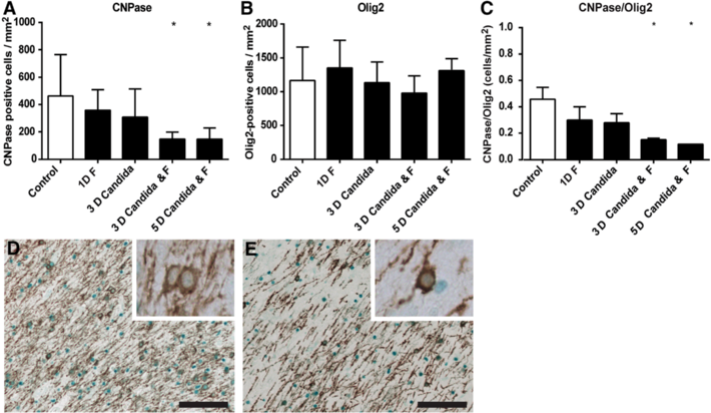
Myelin disturbances are accompanied by a loss of mature oligodendrocytes. A significant decrease (*p < 0.05) of the ratio between CNPase-positive mature oligodendrocytes and total number of cells of oligodendroglial lineage (Olig2-positive) was observed following 3-day *C. albicans* and fluconazole (F) treatment and 5-day *C. albicans*-exposed groups and fluconazole treatment compared to controls. **a**, **b** graphical representation of the CNPase (**a**) and Olig2 (**b**) data; (**c**) graphical representation of the CNPase/Olig2 ratio; **d**–**e** representative figures of CNPase/Olig2 double stain in (**d**) controls and (**e**) 5-day *C. albicans*-exposed animals indicating an apparent loss of CNPase-positive cells. Images taken at ×200 magnification (*insert* at ×400 magnification), *scale bar* = 100 μm

There was reduced MBP immunoreactivity within regions of overt microglial activation in the 3-day *C. albicans*-exposed animals. In addition, myelin texture was disturbed in all *C. albicans*-exposed animals whereas no myelin disturbances were observed in control animals (Fig. 4i–l). The apparent loss of MBP IR and altered texture of myelin prompted us to further investigate the ratio between mature and total oligodendroglial lineage cells. Double staining for CNPase and Olig2 revealed a significant decrease of CNPase-positive cells in the 3-day *C. albicans/*fluconazole and 5-day *C. albicans/*fluconazole groups whereas the number of Olig2-positive cells remained identical, thereby resulting in a decreased ratio of mature vs. total oligodendrocytes in these animals (Fig. 6a–e).

### Antenatal exposure to *C. albicans* did not result in neuronal degeneration

In all experimental groups, no Fluoro-Jade C-positive neurons were detected throughout the brain sections (data not shown).

### MPO and CD3

MPO- and CD3-positive cells were sporadically detected in the cerebral vasculature of the choroid plexus and meninges. No MPO- and CD3-positive cells were found in the cerebral white matter. No differences were seen between all experimental groups (Additional file 1: Figure S1).

### Ki67 and caspase-3

**Fig. 7:**
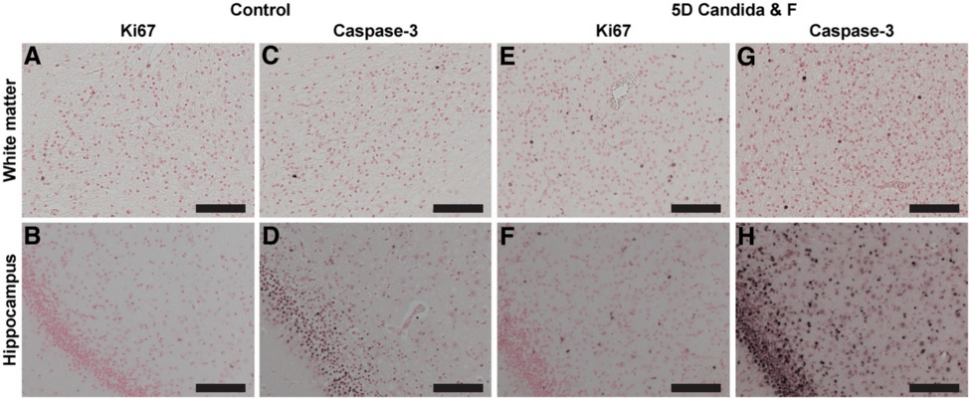
Antenatal exposure to *C. albicans* resulted in increased proliferation and apoptosis in the subcortical white matter and hippocampus. A significant increase (*p < 0.05) of Ki67- and caspase-3-positive cells were observed following 5-day *C. albicans* with fluconazole treatment in the white matter (**e**, **g**) and hippocampus (**f**, **h**) compared to controls (**a**–**d**). Images taken at ×200 magnification, *scale bar* = 100 μm

An increase in Ki67- and caspase-3-positive cells was found in the white matter and hippocampus of the 5-day *C. albicans*/fluconazole group compared to controls (Fig. 7a–h, Table 2). Moreover, in the periventricular white matter, an increase in caspase-3-positive cells was observed.

**Table 2:** Ki67- and caspase-3-positive cells

	Control	1-day fluconazole	3-day *C. albicans*	3-day *C. albicans* and fluconazole	5-day *C. albicans* and fluconazole
Ki67 + cells/mm^2^					
White matter	11.2 (7.2)	41.6 (38.7)	21.9 (16.4)	54.7 (71.0)	56.0 (31.1)*
Periventricular white matter	7.6 (9.1)	14.4 (11.2)	17.6 (14.2)	8.6 (10.7)	20.9 (18.9)
Hippocampus	5.6 (4.2)	16.8 (13.6)	22.2 (27.5)	13.1 (13.0)	33.4 (27.0)*
Caspase-3 + cells/mm^2^					
White matter	12.8 (13.6)	14.1 (11.1)	23.7 (33.2)	16.9 (6.8)	40.2 (22.6)*
Periventricular white matter	26.1 (11.4)	35.4 (24.4)	93.4 (110.2)	46.4 (29.1)	155.1 (22.4)*
Hippocampus	364.3 (175.3)	264.4 (19.6)	443.2 (198.8)	313.4 (117.0)	726.3 (317.9)*

Ki67- and caspase-3-positive cells expressed as mean (±SD)

*p < 0.05 vs. control

## Discussion

The main finding of this study is that 3 days of intra-amniotic exposure to *C. albicans* resulted in increased microglial and astrocyte activation with subsequent focal white matter disturbances in the preterm ovine brain. At this stage, *C. albicans*-positive CSF cultures were detected without *C. albicans*-positive immunostainings of the brain. This may indicate that cerebral inflammation and injury in the acute phase after intra-amniotic *C. albicans* delivery is not caused by invasion of *C. albicans* in the brain parenchyma [25, 26]. This concept is supported by the absence of neutrophils in the brain parenchyma since neutrophils are known to aggravate disruption of the blood-brain barrier promoting further invasion of *C. albicans* in the brain [25]. However, we cannot rule out the possibility that low titers invaded the brain, since the initial phase of *C. albicans* invasion does not require a disruption of the blood-brain barrier and such low fungal burden of the brain cannot be detected by histology or immunofluorescence [15, 25].

Since *C. albicans* was not detected in the fetal brain, we considered that severe systemic immune activation, as shown in the present and previous studies [16, 17], was (primarily) responsible for the acute changes observed in the fetal brain 3 day post-*C. albicans* infection of the amniotic cavity for the following reasons: Pro-inflammatory cytokines, such as IL-6, contribute to activation of the endothelium of the blood-brain barrier, which in turn activate adjacent microglia and astrocytes, ultimately resulting in a cerebral inflammatory response [27–31]. In line with this, systemic inflammation is known to be causally involved in the induction of white matter injury—as seen in our model [32, 33]. In addition, IL-6 was shown to be involved in the inhibition of neurogenesis [28] and the development of hyper-excitable neurological conditions including epilepsy, psychoses, anxiety, and autism spectrum disorders in experimental models [34].

Notably, increased circulatory IL-6 concentrations were not always paralleled by positive blood cultures in our study. This finding can be explained by earlier studies [35, 36] in which the presence of a pro-inflammatory trigger in the amniotic fluid caused systemic inflammation without migration of micro-organisms or their components into the fetal circulation.

Intra-amniotic *C. albicans* caused a heterogeneous pattern of cerebral immune activation as illustrated by increased IBA-1 immunoreactivity in all *C. albicans-*exposed animals. These regions of overt microgliosis were strongly associated with white matter injury within these specific regions. Moreover, expression of CD68, which is important for the phagocytic activity of macrophages/microglia, was increased at 5 days post exposure and might play a prominent role in the clearance of debris including injured mature oligodendrocytes and myelin [37].

White matter injury in this study was illustrated by reduction of MBP IR at 3 days post *C. albicans* exposure, disrupted myelin texture in all *C. albicans-*exposed animals and a deficit of mature oligodendrocytes which was most prominent in the 5-day *C. albicans*/fluconazole group. This white matter injury is considered to be the result of activated microglia as seen in our study, which produce cytokines, free radicals, nitric oxide, and excitotoxic amino acids, all detrimental for oligodendrocytes and myelin [7, 38]. In addition, the most prominent loss of mature oligodendrocytes in the 5-day *C. albicans*/fluzonazole group is most likely the result of clearance of these cells by phagocytizing microglia as previously stated. This deficit was paralleled by replenishment of the oligodendrocyte pool via two mechanisms. First, oligodendrocyte progenitors could migrate to the site of injury [39, 40]. Second, increased oligodendrocyte numbers could be the consequence of increased proliferation [39, 41], which would be consistent with our Ki67 data. However, increased Ki67-positive cells do not exclusively identify oligodendrocyte proliferation but could also comprise other cell types. In particular, the increase of Ki67-positive cells could also, in part, account for microglial proliferation, since we observed an increase of microglia within these regions.

Nonetheless, the observed replenishment of oligodendrocytes is considered to be initiated as compensation for the observed reduction of mature oligodendrocytes [39, 41]. It is tempting to speculate that these progenitors might fail to mature into a functional myelin-producing oligodendrocyte since this phenotype has previously been reported in experimental models of perinatal stress [42–44] and in human neonates with periventricular leukomalacia [39]. Finally, such oligodendrocyte maturation arrest correlates with motor deficit characteristic for cerebral palsy [38, 44, 45]. In conjunction with white matter injury, grey matter injury is increasingly recognized as a contributor to adverse neurodevelopmental outcome [35]. However, in our study, no degenerating neurons were observed [22, 23]. Although it is not likely that neurodegeneration is already pertinent in the 5-day *C. albicans*/fluconazole group, we cannot exclude the possibility that the absence of Fluoro-Jade staining at 5 days post exposure is the result of already degenerated and dead neurons. In addition, cerebral palsy also comprises cognitive impairments [7, 8] for which we analyzed the hippocampus in which a similar inflammatory response was observed which is consistent with our previous results [21, 46].

We previously reported that intra-amniotic *C. albicans* rapidly colonizes the amniotic fluid, causing inflammation of the fetal skin and lungs, and progresses into a fetal systemic inflammatory response causing fetal death within 5 days after exposure [16, 17]. In the present study, we show that fluconazole administration eradicated *C. albicans* from the CSF and reduced systemic inflammation. Importantly, reduction of the fungal burden and inhibition of systemic immune activation following fluconazole treatment was not accompanied by attenuation of cerebral inflammation and injury. Multiple studies have demonstrated that cerebral inflammation acquired during early fetal development continues postnatally and might even persist into adulthood [47, 48]. Therefore, we consider that the observed cerebral inflammatory response following intra-amniotic *C. albicans* exposure is a persistent reaction that is initiated by peripheral immune activation. Although fluconazole treatment did not protect against structural cerebral injury in this acute phase, the observed inhibition of systemic immune activation might be clinically relevant, since prolonged and aberrant systemic immune activations are known to induce blood-brain barrier disruption, thereby facilitating cerebral invasion of *C. albicans* and substantially aggravating cerebral injury resulting in increased morbidity and mortality [26]. Collectively, previous and current findings suggest that timing of infection and the start of antifungal treatment regimen are important in the initiation, maintenance, and possible resolution of neuroinflammation and structural injury in the fetal brain. The current study provides important insight in the sequelae of events that contribute to induction of adverse outcomes of the premature brain following intra-amniotic *C. albicans* infection. In addition, this study points out that fluconazole treatment should be started immediately after intra-amniotic exposure to *C. albicans* considering the narrow therapeutic time window to reduce morbidity and mortality. Therefore, further research should focus on timing, frequency, and dosing of antifungals in the presence or absence of immunomodulatory treatment [49].

Fluconazole treatment was not associated with adverse effects in our study, which is in line with the findings of Maneenil and colleagues in the same model [16] and studies demonstrating that teratogenic effects of fluconazole are exclusively seen when used in the first trimester at higher doses and after multiple gifts [50]. The administered fluconazole dose in this study mimics the amount used in systemic neonatal candidiasis in which no adverse long-term brain pathology and behavioral deficits are currently described [50]. In addition, clinical *in utero* administration of fluconazole prolonged pregnancy in two cases of intra-amniotic *C. albicans* infection without adverse treatment effects on the fetus [13].

We note that our study has several limitations. We only tested one dose and frequency of fluconazole administration. In addition, we were not able to correlate histological changes to functional neurological outcomes. However, several studies previously established an association between endotoxin-induced chorioamnionitis and functional electro-encephalogram (EEG) changes [43, 51, 52].

## Conclusions

We have shown that intra-amniotic exposure to *C. albicans* results in an acute systemic and neuroinflammatory response with concomitant white matter injury. Although systemic immune activation was significantly inhibited following fluconazole treatment, modulation of the cerebral inflammatory response and prevention of concomitant white matter injury was not found.

This study forms the essential basis for follow-up studies in which the timing, frequency, and dosing of antifungal treatment must be explored, and alternative/additional neuroprotective treatment options including immunomodulatory interventions can be tested.

## Additional file

Additional file 1: Figure S1. Antenatal exposure to C. albicans does not result in infiltration of peripheral immune cells. (A–B) representative pictures of CD3-positive T lymphocytes in the meninges of (A) controls and (B) 5-day *C. albicans*/fluconazole animals. (C–D) representative pictures of MPO-positive neutrophils in the choroid plexus of (C) controls and (D) 5-day *C. albicans*/fluconazole animals. (TIF 20.7 mb)

## Abbreviations

BSA: bovine serum albumin
CD3: cluster of differentiation 3
CD68: cluster of differentiation 68
CFU: colony-forming units
CNPase: 2',3'-cyclic-nucleotide 3'-phosphodiesterase
CSF: cerebrospinal fluid
DAB: 3,3'-diaminobenzidine
EEG: electro-encephalogram
ELISA: enzyme-linked immunosorbent assay
F: fluconazole
FIRS: fetal inflammatory response syndrome
GFAP: glial fibrillary acidic protein
H&E: hematoxylin and eosin
HRP: horseradish peroxidase
IA: intra-amniotic
IBA-1: ionized calcium-binding adaptor molecule 1
IL-6: interleukin-6
IP: intra-peritoneal
IR: immunoreactivity
MBP: myelin basic protein
MPO: myeloperoxidase
Olig2: oligodendrocyte transcription factor 2
PAS: periodic acid-Schiff
PBS: phosphate-buffered saline
PBST: Tween-20 in phosphate-buffered saline
PVWM: periventricular white matter
SEM: standard error of the mean
TMB: 3,3',5,5'-tetramethylbenzidine
WM: white matter
