# Supplementary figures and images for: Neuroinflammation and structural injury of the fetal ovine brain following intra-amniotic Candida albicans exposure
Source: J Neuroinflammation. 2016 Feb 2;13:29. doi: 10.1186/s12974-016-0492-z (PMC4739103; doi:10.1186/s12974-016-0492-z)

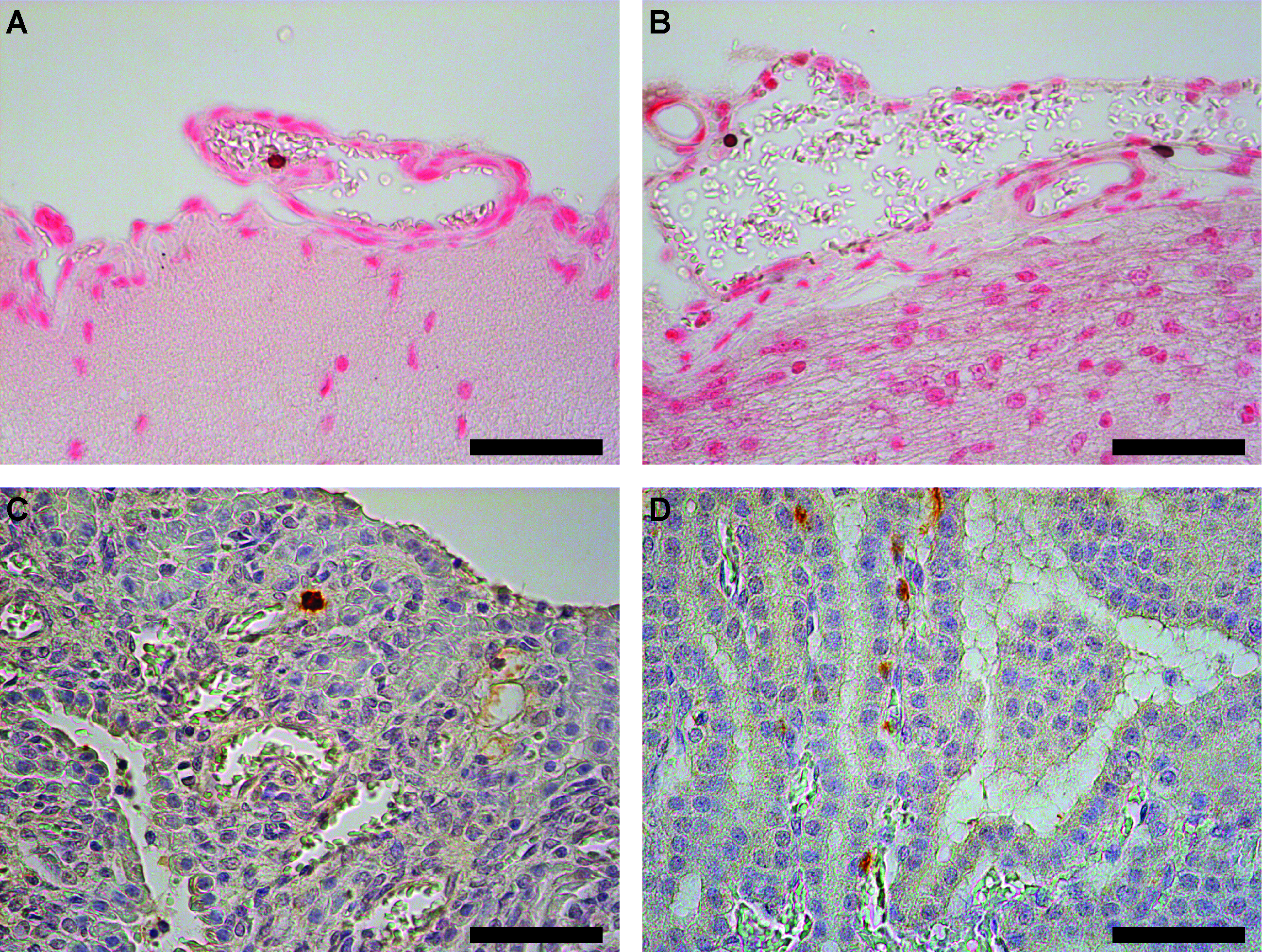

Supplement: Additional file 1: Figure S1. — Antenatal exposure to C. albicans does not result in infiltration of peripheral immune cells. (A–B) representative pictures of CD3-positive T lymphocytes in the meninges of (A) controls and (B) 5-day C. albicans/fluconazole animals. (C–D) representative pictures of MPO-positive neutrophils in the choroid plexus of (C) controls and (D) 5-day C. albicans/fluconazole animals. (TIF 20.7 mb) [file 12974_2016_492_MOESM1_ESM.tif]
